# Improving the Virtual Screening Ability of Target-Specific Scoring Functions Using Deep Learning Methods

**DOI:** 10.3389/fphar.2019.00924

**Published:** 2019-08-22

**Authors:** Dingyan Wang, Chen Cui, Xiaoyu Ding, Zhaoping Xiong, Mingyue Zheng, Xiaomin Luo, Hualiang Jiang, Kaixian Chen

**Affiliations:** ^1^Drug Discovery and Design Center, State Key Laboratory of Drug Research, Shanghai Institute of Materia Medica, Chinese Academy of Sciences, Shanghai, China; ^2^College of Pharmacy, University of Chinese Academy of Sciences, Beijing, China; ^3^School of Life Science and Technology, ShanghaiTech University, Shanghai, China

**Keywords:** virtual screening, target-specific scoring function, deep learning, drug discovery, DUD-E

## Abstract

Scoring functions play an important role in structure-based virtual screening. It has been widely accepted that target-specific scoring functions (TSSFs) may achieve better performance compared with universal scoring functions in actual drug research and development processes. A method that can effectively construct TSSFs will be of great value to drug design and discovery. In this work, we proposed a deep learning–based model named DeepScore to achieve this goal. DeepScore adopted the form of PMF scoring function to calculate protein–ligand binding affinity. However, different from PMF scoring function, in DeepScore, the score for each protein–ligand atom pair was calculated using a feedforward neural network. Our model significantly outperformed Glide Gscore on validation data set DUD-E. The average ROC-AUC on 102 targets was 0.98. We also combined Gscore and DeepScore together using a consensus method and put forward a consensus model named DeepScoreCS. The comparison results showed that DeepScore outperformed other machine learning–based TSSFs building methods. Furthermore, we presented a strategy to visualize the prediction of DeepScore. All of these results clearly demonstrated that DeepScore would be a useful model in constructing TSSFs and represented a novel way incorporating deep learning and drug design.

## Introduction

Structure-based drug design (SBDD) has been widely used in industry and academia (Andricopulo et al., 2009; Morrow et al., 2012). There are three main categories of tasks for SBDD methods: virtual screening, *de novo* drug design, and ligand optimization. Virtual screening generally refers to the process of identifying active compounds among molecules selected from a virtual compound library. By utilizing the three-dimensional information of proteins, structure-based virtual screening is believed to be more efficient than traditional virtual screening methods. The key factor for guaranteeing the success of structure-based virtual screening is the quality of scoring functions. Theoretically, a scoring function is capable of predicting the binding affinity of a protein–ligand complex structure, and thus can be used for predicting the binding pose of a ligand or screening a virtual compound library to find potential active compounds.

Classic scoring functions can be divided into three categories: force field–based, knowledge-based, and empirical (Liu et al., 2017). For a long time, researchers have found that machine learning and deep learning methods had an excellent performance in helping constructing different kinds of scoring functions. Especially recently, convolutional neural network (CNN) utilizing the structural information of protein–ligand complexes has shown promise in predicting binding affinity and virtual screening (Ragoza et al., 2017; Stepniewska-Dziubinska et al., 2018). A deep learning model constructed using CNN by Imrie et al. represented the state-of-the-art on several virtual screening benchmarks (Imrie et al., 2018). However, the authors also found that fine-tuning a general model on subsets of a specific protein family resulted in a significant improvement. This reflects the fact that no single scoring function is suitable for every target. Moreover, in practice, a medicinal chemist is usually concerned about only one target at a time and hope that the scoring function he uses has the best performance on this target. The most common and direct way to address this issue is to build a target-specific scoring function (TSSF) for the specific target. TSSFs have been widely used in virtual screening campaign and proved to be useful in variable kinds of important drug targets including kinases (Xu et al., 2017; Berishvili et al., 2018) and GPCRs (Kooistra et al., 2016).

Based on the fact mentioned above, it is of great value to design a method that can effectively construct TSSFs. Several methods have been proposed to address this problem. In 2005, Antes et al. presented a model called Parameter Optimization using Ensemble Methods (POEM) which applied the design of experiments (DOE) approach and ensemble methods to the optimization of TSSFs in molecular docking (Antes et al., 2005). They fitted FlexX and ScreenScore to the kinase and ATPase protein classes and got a promising result. In 2010, Xue et al. developed a kinase-specific scoring function named kinase-PMF in order to score ATP-competitive inhibitors (Xue et al., 2010). Their work showed that TSSFs achieved better performance compared with general scorings. In 2011, Li et al. proposed a scoring function building strategy named SVM-SP based on support vector machine (SVM) (Li et al., 2011). They tailored SVM-SP to each target in the test set and found that it outperformed many other scoring functions including Glide. In 2015, Wang et al. introduced a strategy named TS-Chemscore to build TSSFs based on a known universal scoring function by a regression process on energy contributions (Wang et al., 2015). In 2017, Yan et al. used a residue-based interaction decomposition method with SVM to develop a target-specific discrimination model called protein–ligand empirical interaction components-SVM (PLEIC-SVM) (Yan et al., 2017). Their results showed that PLEIC-SVM was a useful tool in filtering the docking poses.

Here, we introduce a deep learning–based method named DeepScore used for constructing TSSFs. The purpose of DeepScore is rescoring the docking poses generated from docking software like Glide. DeepScore uses the scoring model of PMF scoring function, where the score for a protein–ligand complex is derived from the sum of protein–ligand atom pair-wise interactions within a distance range. The score for a single protein–ligand atom pair is calculated using a fully connected neural network. Since consensus scoring methods have shown to be useful in improving the performance considering the results from several different models (Teramoto and Fukunishi, 2008; Ericksen et al., 2017), we further proposed DeepScoreCS by combining the results of DeepScore and Glide Gscore together. The directory of useful decoys–enhanced (DUD-E) was used as the benchmark to quantitatively assess the model. 12 metrics were calculated and used for making comparison between Gscore, DeepScore, DeepScoreCS, and some other TSSF models reported by previous studies.

## Materials and Methods

### Data Preparation

The directory of useful decoys–enhanced (DUD-E) benchmarking set (Mysinger et al., 2012) was used for training and evaluating the model. DUD-E is a data set designed for helping benchmark docking software and scoring functions. There are 102 targets in DUD-E. Each target is provided with 224 active ligands and 13,835 decoys on average. DUD-E has been widely used for evaluating the virtual screen ability of scoring functions (Chaput et al., 2016; Ericksen et al., 2017; Ragoza et al., 2017; Yan et al., 2017; Imrie et al., 2018). Although it has been reported by some literature that there exists noncausal bias in DUD-E (Sieg et al., 2019), we still use it to evaluate our model since there is no better data set so far.

The first step is to generate docking poses for actives and decoys. We noticed that, in other similar work, a variety of docking methods were used in this step, including Glide (Yan et al., 2017), AutoDock Vina (Imrie et al., 2018), DOCK (Pereira et al., 2016), PLANTS (Kurkinen et al., 2018), and so on. Even using the same docking program, sometimes different docking protocols were adopted (Chaput et al., 2016; Yan et al., 2017). It should be emphasized that, strictly speaking, only the rescoring results from the same docking poses are comparable.

Since the ligands in DUD-E have been assigned appropriate protonation states, we followed the approach in (Chaput et al., 2016) that ligands were used without any modified. Receptors were prepared with protein preparation wizard from Schrodinger suit (Schrödinger, LLC, New York, NY, 2015-2). Ligands were docked using Glide (Friesner et al., 2006) in SP mode with default options.

### Descriptors and Model

Through data preparation step, the best poses ranked by Gscore were selected for actives and decoys. To rescore the docking poses from Glide, we utilized the form of the potential of mean-force (PMF) scoring function (Muegge and Martin, 1999) to calculate the score for each protein–ligand complex. In PMF scoring function, the score for a complex is defined as the sum of overall protein–ligand atom pair-wise interactions within a specific cutoff radius:

(1) $$\begin{array}{ll} PMFScore_{complex} & =\;\sum_{i}\sum_{j}A\left(i,j,distance_{ij}\right)\;for\;distance_{ij\;}\\ & <cutoff\;distance \end{array}$$

where i is the ligand atom, j is the receptor atom, distance_ij_ is the distance between atom i and atom j, and A is the function used for calculating the PMF between atom i and atom j.

In Pafnucy (Stepniewska-Dziubinska et al., 2018), a structure-based CNN model, 19 features were used for describing an atom. In DeepScore, almost same features but with minor modifications were used (see **Table 1**). The features included the information of atom type, hybridization state, heavy valence, hetero valence, partial charge, and whether the atom was aromatic/hydrophobic/hydrogen-bond donor/hydrogen-bond acceptor/in a ring. Heavy valence and hetero valence were represented as one-hot vectors in DeepScore instead of integers in Pafnucy.

**Table 1:** Atom features used in DeepScore.

Atom feature name	Feature length	Features
Type	9	B, C, N, O, P, S, Se, halogen, and metal
Hybridization	4	1, 2, 3, other
Heavy valence^a^	4	1, 2, 3, other
Hetero valence^b^	5	0, 1, 2, 3, other
Partial charge	1	Value
Hydrophobic	1	1 (True) or 0 (false)
Aromatic	1	1 (True) or 0 (false)
Hydrogen-bond donor	1	1 (True) or 0 (false)
Hydrogen-bond acceptor	1	1 (True) or 0 (false)
Ring	1	1 (True) or 0 (false)

^a^The number of bonds with other heavy atoms.

^b^The number of bonds with other heteroatoms.

**Figure 1:**
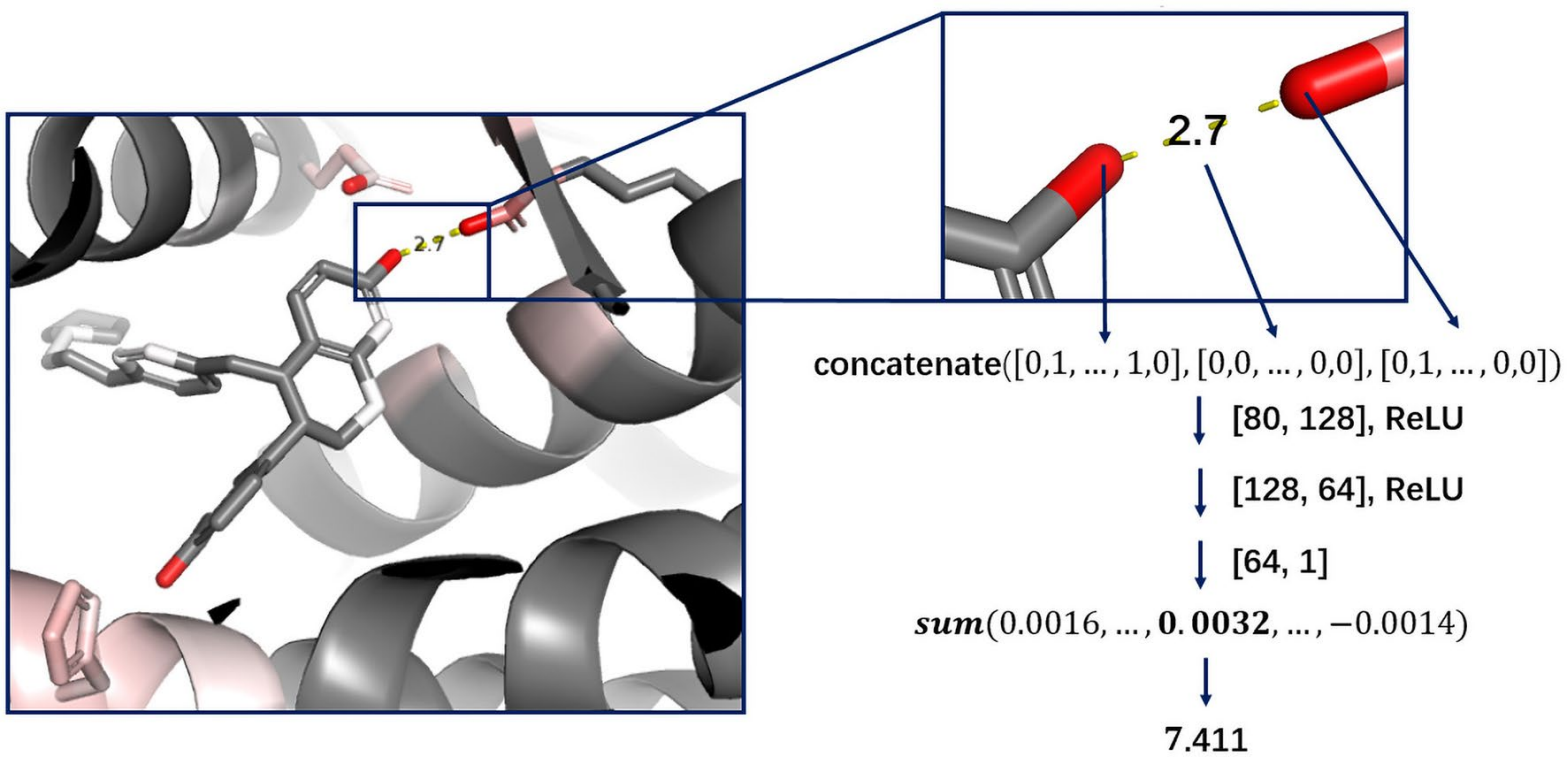
Workflow of DeepScore model construction.

Cutoff distance was changed to an accepted distance range in DeepScore. For each complex, atom pairs between 2 and 8 Å were sorted in the ascending order of length, and only 500 shortest pairs were taken into consideration. Distance was also discretized with bins equally distanced by 0.025 Å between 2 and 8 Å. The feature for a protein–ligand atom pair was comprised of the concatenation of the ligand atom feature vector, the protein atom feature vector, and the one-hot-encoded distance, which made the length of an atom pair feature 80 bins long (Eq. 2-1). The score for an atom pair (i-j) was calculated as Eq. 2-2 using a 2-hidden layer fully connected network. The sizes of weight matrix W_1_, W_2_, and W_3_ were 80×128,128×64,64×1, respectively. b_1_, b_2_, and b_3_ were biases. Rectified linear unit (ReLU) was used as activation function. Final score, or DeepScore, for a protein–ligand complex was calculated as Eq. 2-3. In Eq. 2-3, i and j refer to the ligand atom and the receptor atom respectively. All calculated scores of selected protein–ligand atom pairs were summed up to determine the final score. Overview of the workflow is also shown in **Figure 1**.

(2-1) $$Feature_{ij}=concatenate\left(Feature_{i},\;Distance_{ij},Feature_{j}\right)$$

(2-2) $$DeepScore_{ij}=W_{3}\left(\text{ReLU}\left(W_{2}\left(ReLU\left(W_{1}Feature_{ij}+b_{1}\right)\right)+b_{2}\right)\right)+b_{3}$$

(2-3) $$DeepScore_{complex}=\sum_{i-j}DeepScore_{ij}\;for\;selected\;atom\;pair\;i-j\;$$

### Loss Function

In deep learning processes, the usual practice while dealing with a two possible classification problem is to put two units in the output layer and transform the outputs using softmax function. The outputs, which represent the probability of classes 0 and 1, respectively, are then used for calculating the loss with cross entropy loss or other loss functions. However, in practice, we found that the cross entropy loss function did not apply to our model very well. We tried some other loss functions and found that modified Huber loss (Eq. 3) (Zhang, 2004) was more suitable. The formula of modified Huber loss is shown in Eq. 3, where f(x) refers to the output of the model and y refers to the label (1 for actives and -1 for decoys). It should be noted that, unlike general scoring functions, the possible scoring range of DeepScore is the entire real number filed. A score greater than zero indicates that the model considers the compound to be active, whereas a score less than zero is inactive. Another important point is that scores between different targets are not comparable.

(3) $$\text{L}\left(\text{y},\text{ f(x)}\right)=\;\{\begin{array}{cc} {\left[\max\left(0,\;1-yf\text{(}x\text{)}\right)\right]}^{2}\; & for\;yf(x)\geq -1,\\ -4yf\text{(}x\text{)} & \;otherwise.\; \end{array}$$

### Training

Five-fold cross validation test was performed on each target in DUD-E. For each target, the whole data set was split into five parts at first. Within each fold, three parts were used as training set, one part as validation set, and one part as test set. The order of [(training set)/validation set/test set], we used during cross validation was ([1,2,3]/4/5), ([2,3,4]/5/1), ([3,4,5]/1/2), ([4,5,1]/2/3), and ([5,1,2]/3/4). Early stopping strategy was used for avoiding overfitting. For each training epoch, the area under the curve of precision recall curve (PRC-AUC) on validation set was calculated. If the performance did not improve within eight epochs, training was stopped, and the best model was saved and evaluated on test set. Mean value of the metrics of five folds on test set was calculated and used as the performance of the model. To make it fair, the performance of Gscore was also calculated in the same way.

It should be noticed that there existed a dramatic class imbalance in our data sets as the number of decoys was almost 50 times of that of actives. To overcome this problem, we adopted the random undersampling strategy. Over an epoch, we did not use the whole training set to train the model. Instead, parts of decoys were randomly selected out to make sure that the number of actives and decoys was the same in an epoch. The reason why we chose undersampling was that, compared with other methods like oversampling, the training procedure using this strategy was significantly faster.

Our model was implemented using PyTorch 1.0 (https://pytorch.org/) in python. Each model was trained using Adam optimizer with a batch size of 32, a learning rate of 0.001, and a weight decay of 0.001.

### Evaluation Metrics

The area under the curve of receiver operating characteristic curve (ROC-AUC), the PRC-AUC, enrichment factor (EF), and ROC Enrichment Factor (ROC-EF) were calculated for each fold in order to evaluate the performance of the model. ROC-AUC is a traditional metric for assessing the performance of a classification model. However, under the circumstance that the number of negative samples is obviously larger than the number of positive, like our mission, PRC-AUC is usually a more appropriate choice to replace ROC-AUC since ROC-AUC may not reflect the early enrichment ability of the model (Truchon and Bayly, 2007). EF is the fraction of actives within a certain percentage of ranking list divided by the fraction in whole data set. Because the way of calculating EF simulates actual virtual screening scenarios where only a small fraction of ligands are picked out to carry out biological test, EF is one of the gold standards used for evaluating ranking ability of scoring functions. ROC-EF is another metric recommended by Jain et al. (Jain and Nicholls, 2008) to quantify early enrichment. It refers to the rate of true-positive rate (TPR) to false-positive rate (FPR) at certain FPR. Both EF and ROC-EF were calculated at five different levels of percentage: 0.5%, 1%, 2%, 5%, and 10%. Thus, there were in all 12 metrics for evaluating the models.

### Consensus Scoring

When the correlation between the statistical errors of multiple models is low, combining the predicted values of these models in a certain way usually performs better than any single one model. This is the basic idea of ensemble learning (Dietterich, 2000). We adopted this strategy and used Eq. 4 to calculate DeepScoreCS for a complex. In Eq. 4, c is a coefficient that can be adjusted. More details will be showed and discussed in Results and Discussion part.

(4) DeepScoreCS=DeepScore×c+Gscore×(1−c) , 0≤c≤1

## Results and Discussion

### Model Architecture

Deep learning models are usually regarded as black boxes since the information of which features that are important can hardly be interpreted from the model. Although CNN based scoring functions, like Pafnucy from which the atom features of DeepScore were borrowed, have achieved state-of-the-art performance in benchmark test, and become the representative of deep learning–based scoring functions, treating the whole protein–ligand complex as a 3D picture is still counterintuitive. Thus, in consideration of interpretation, we chose to reform the classic PMF scoring function. The neural network in DeepScore is only used to facilitate the learning of atom-pair potentials; meanwhile, the overall framework of PMF scoring function is preserved. DeepScore is able to directly give the score of each atom pair, which makes the model’s output easy to explain. To the best of our knowledge, DeepScore is the first model to use this framework.

### Glide Screening

**Figure 2:**
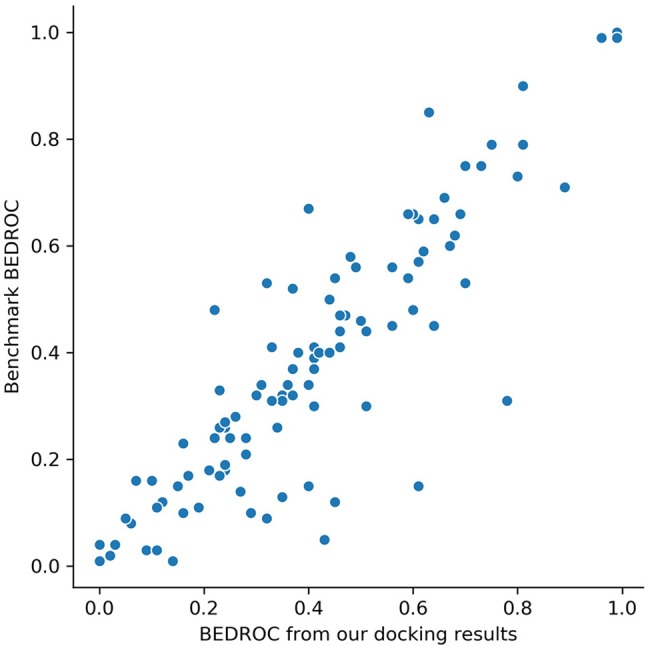
BEDROC scores (α=80.5) on 102 targets of our screening results *versus* the results from benchmark (Chaput et al., 2016). Each dot represents a target.

Glide docking results are provided in **Table S1**. For DUD-E data set, the mean value of ROC-AUC gained from Glide was 0.82, which showed a significant better screening ability compared with other docking software, like AutoDock Vina (0.703) (Imrie et al., 2018). To ensure the reliability of docking poses, we compared Boltzmann-enhanced discrimination ROC (BEDROC, α=80.5) of our results with (Chaput et al., 2016) on each target, since we used the same docking software and similar docking protocol with them. The scatter plotting is shown in **Figure 2**. Our results showed a high correlation with (Chaput et al., 2016), which ensured that the docking poses are credible.

### DeepScore

**Figure 3:**
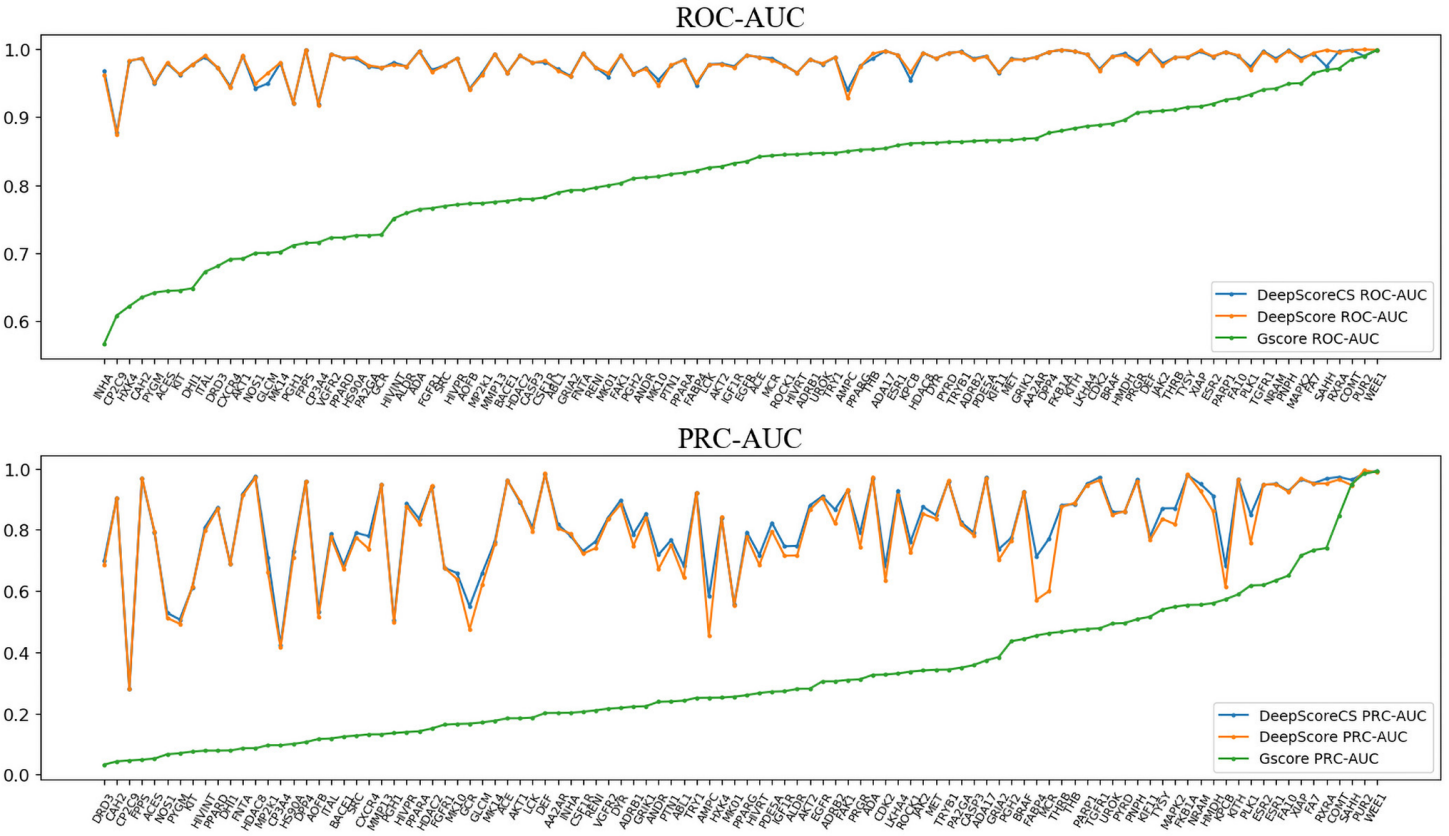
ROC-AUC (upper panel) and PRC-AUC (lower panel) of cross validation performance on each target. Targets are sorted by the performance of Gscore.

ROC-AUC, PRC-AUC, EF (0.5%, 1%, 2%, 5%, and 10%), ROC-EF (0.5%, 1%, 2%, 5%, and 10%) of Gscore, and DeepScore on all 102 targets were calculated (see **Figure 3**, **Table S2** and **Table S3**). **Figure 3** shows that DeepScore performs better than Gscore significantly. DeepScore had an excellent performance on ROC-AUC where all the targets showed an improvement *versus* Gscore. The mean values of 12 metrics were all increased by using DeepScore, as shown in **Table 2**.

**Table 2:** Average performance of Gscore, DeepScore, and DeepScoreCS on DUD-E data set.

	Gscore	DeepScore		DeepScoreCS	
	Value	Value	Better-1^a^	Value	Better-2^b^
ROC-AUC	0.817	0.979	102	0.978	49 81 51 65 60 40 20 66 47 42 35 28
PRC-AUC	0.317	0.796	100	0.814	
EF0.5%	30.625	55.275	94	57.149	
EF1%	24.335	52.218	98	53.658	
EF2%	17.203	39.716	100	40.075	
EF5%	9.122	18.200	102	18.200	
EF10%	5.573	9.472	101	9.448	
ROC-EF0.5%	51.522	148.948	100	151.986	
ROC-EF1%	31.239	81.614	102	82.164	
ROC-EF2%	18.689	43.320	102	43.498	
ROC-EF5%	9.423	18.417	101	18.365	
ROC-EF10%	5.680	9.500	101	9.484	

^a^ Better-1 column refers to the number of targets where DeepScore outperforms Gscore.

^b^ Better-2 column refers to the number of targets where DeepScoreCS outperforms DeepScore.

**Figure 4:**
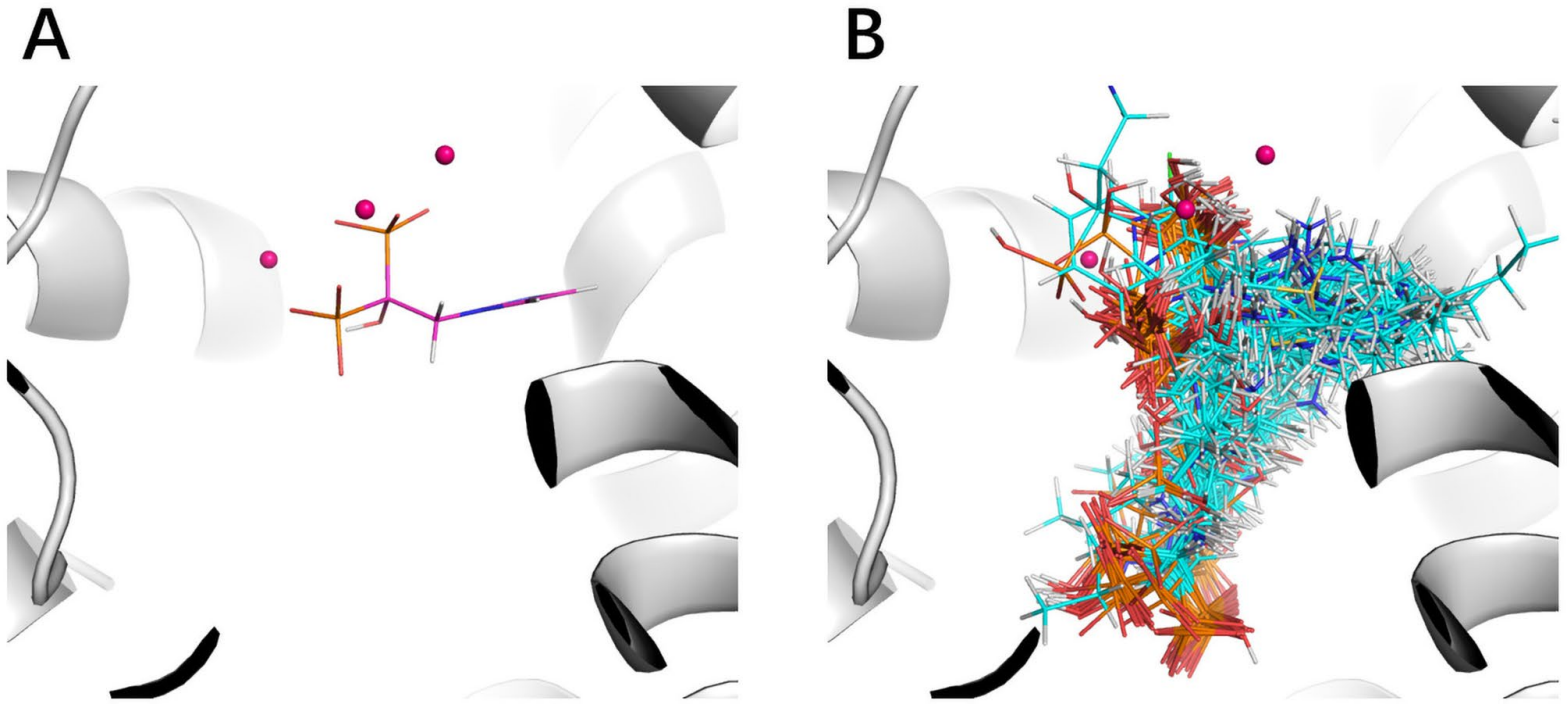
The binding site of FPPS (PDB ID 1zw5). **(A)** Crystal structure ligand. **(B)** Superposition of all the docking poses of actives.

The improvement of performance on some targets was obvious. For example, for target FPPS (farnesyl diphosphate synthase), the ROC-AUC of Gscore was 0.54, which indicated that Gscore just randomly scored actives and decoys on FPPS. On the other side, ROC-AUC of DeepScore was 1.00 which demonstrated that DeepScore could almost perfectly separate actives and decoys. Similar situation also arose in (Ragoza et al., 2017). In this study, authors found that AutoDock Vina got a worse-than-random ROC-AUC of 0.29 on FPPS, while the “DUD-E only model” they trained also performed excellently with a ROC-AUC of 0.98. The authors supposed that the reason why AutoDock Vina performed so poorly was that the docking poses of actives were incorrect. However, we found that the wrong docking poses may not be the main reason. As is shown in **Figures 4A, B**, more than half of actives were docked correctly by Glide, where the bisphosphonate group chelated with the magnesium ions, but the performance of Gscore was still very poor. Despite this, we agree with (Ragoza et al., 2017) that the perfect performance of no matter their model or DeepScore was because of simply recognizing the biphosphate group or polarity of molecules since very few decoys possessed phosphorus. It is an extreme example but still highlights two facts. First, DUD-E data set exists the problem of obvious structure differences between decoys and actives, which may result in artificial enrichment during the evaluation of scoring functions and virtual screening methods. Second, TSSFs are more useful than universal scoring functions in the case where the subject is only a single target, because the factors that play a leading role in protein–ligand binding modes in different kinds of targets are different.

### DeepScoreCS

**Figure 5:**
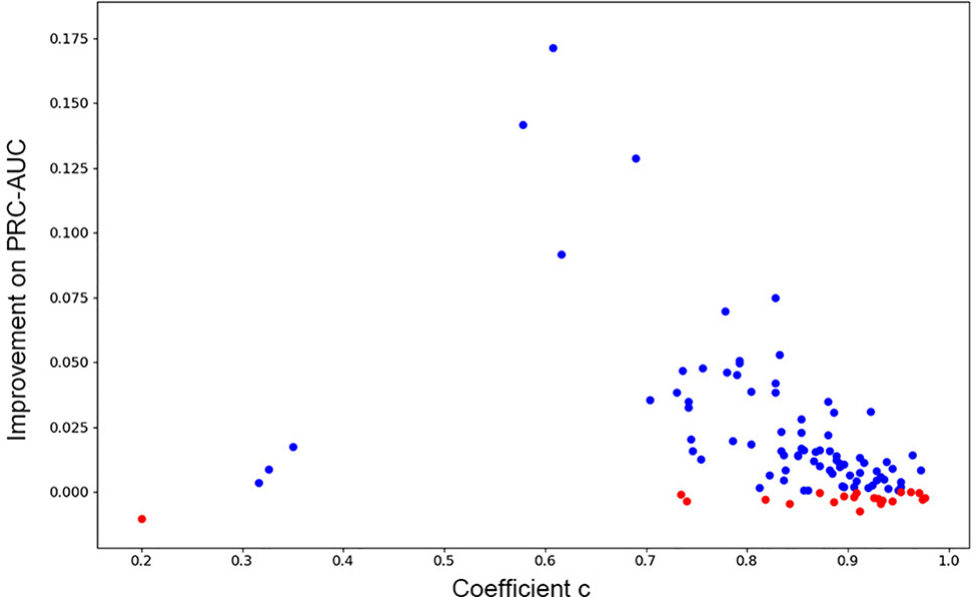
The improvement of PRC-AUC on each target using consensus method. Each point represents a target. Y-axis represents the value of PRC-AUC of DeepScoreCS minus that of DeepScore. Blue dot means that the improvement is positive while red means negative (the performance became worse through consensus method). X-axis represents the mean value of the coefficient c DeepScoreCS used.

As has been mentioned in Methods part, we further investigated if consensus methods could improve the performance of the model in our mission. Eq. 4 was used for calculating the mixture model consensus scores of Gscore and DeepScore. It was important to set an appropriate coefficient c for Eq. 4, and obviously, the best c on each target should be different from each other. Grid searching was used for settling this problem. For each training fold, after the training had stopped, the scores on validation set were determined by the best DeepScore model. Then, different coefficient c from 0 to 1 with step 0.01 was chosen to calculate DeepScoreCS scores on validation set according to Eq. 4. The coefficient c with best PRC-AUC on validation set was used on test set to evaluate the performance of DeepScoreCS. The results are shown in **Table 2**. It can be seen that the improvement of performance by conducting consensus experiment is not obvious. The mean values of PRC-AUC, EF0.5%, EF1%, EF2%, ROC-EF0.5%, ROC-EF1%, and ROC-EF2% increased slightly, while the rest metrics decreased. Most of targets (81/102) got higher PRC-AUC. To investigate whether the performance of the model may actually benefit from consensus methods, we quantitatively examined the improvement of PRC-AUC on each target. The results are presented in **Figure 5**. In **Figure 5**, each point represents a target, X-axis represents the best coefficient c (mean value of five folds) on this target, and Y-axis represents the improvement on PRC-AUC, which is calculated by the PRC-AUC of DeepScoreCS minus that of DeepScore. Targets with higher PRC-AUC are painted blue, and targets with lower PRC-AUC are painted red. It can be noticed that, although on most targets, the impact of consensus strategy was just random perturbation (|Δ*AUC*| < 0.025), no target got a significant decrease on AUC (Δ*AUC* < −0.025). On the other hand, for more than 20 targets, ΔAUC was larger than 0.025. Especially for three targets (AMPC, MCR, and FABP4), the increase of AUC was significant (ΔAUC > 0.1). These results demonstrated that the consensus method was worthy of trying since it would not weaken the performance of the model, and for few targets, the performance would be significantly improved.

### Comparing With Previous Studies

We compared our results with two previous similar studies to check if our model showed better performance.

**Figure 6:**
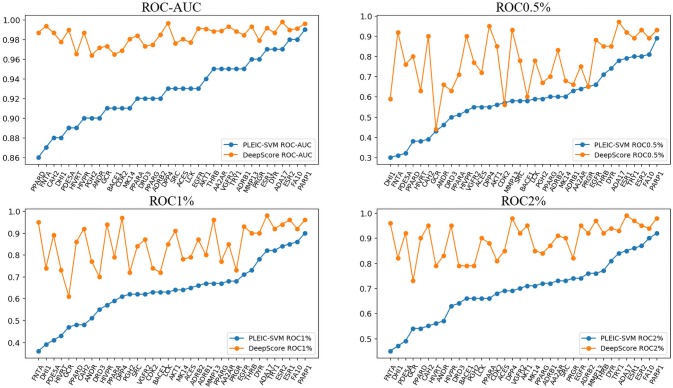
The performance of PLEIC-SVM and DeepScore. Targets are sorted by the performance of PLEIC-SVM.

First, we compared the performance of DeepScore with PLEIC-SVM constructed by Yan et al. (2017). They used 36 targets to train and test their model, so we selected the scores of overlapped targets to make comparison. The results are shown in **Table 3** and **Figure 6**. **Table 3** clearly indicates that DeepScore performed better than PLEIC-SVM. The average ROC-AUC, ROC0.5%, ROC%1, ROC2%, and ROC5% (ROC10% of PLEIC-SVM was not provided) for all 36 targets increased from 0.93, 0.58, 0.64, 0.69, and 0.77 to 0.98, 0.78, 0.85, 0.89, and 0.94, respectively, by using DeepScore. Among these metrics, ROC0.5% is the most important one since the early enrichment ability of scoring functions is paid more attention in the context of virtual screening. **Figure 6** shows that DeepScore outperforms on most of the targets on ROC0.5%. On some targets, such as FNTA, the improvement was dramatic (for FNTA, ROC0.5% increased from 0.31 to 0.92 by using DeepScore). However, for GCR, CDK2, BACE1, and PRGR, DeepScore only got a similar or slightly worse performance.

**Table 3:** Performance comparison between PLEIC-SVM and DeepScore.

	PLEIC-SVM	DeepScore
ROC-AUC	0.93	**0.98**
ROC0.5%^a^	0.58	**0.78**
ROC1%^b^	0.64	**0.85**
ROC2%^c^	0.69	**0.89**
ROC5%^d^	0.77	**0.94**

Performance values of PLEIC-SVM are collected from (Yan et al., 2017). Better results are highlighted in bold.

^a^ ROC0.5% = ROC-EF0.5% / 200.

^b^ ROC1% = ROC-EF1% / 100.

^c^ ROC2% = ROC-EF2% / 50.

^d^ ROC5% = ROC-EF5% / 20.

The workflow of PLEIC-SVM included a process of tuning parameters for SVM model. It should be noticed that, limited by the huge number of targets, we did not perform hyperparameter optimization for every model. In another word, all models were trained under the same set of hyperparameters (learning rate, network structure, etc.). Considering the fact that hyperparameters may significantly affect the performance of machine learning models (also pointed out by (Yan et al., 2017)), it is reasonable to infer that the performance of DeepScore will be further improved by hyperparameter optimization.

We also compared our model with RF-Score. Wójcikowski et al. adopted the same protocol (DUD-E, single target, five-fold cross validations) to evaluate the target-specific virtual screening ability of RF-Score (WÓjcikowski et al., 2017). Descriptors from three versions of RF-Score and ligand binding conformations from three docking programs (AutoDock Vina, DOCK 3.6, and DOCK 6.6) were used for training the model. In all, nine RF-Score models were obtained for testing in their study. The comparison results are presented in **Table 4**. It shows that DeepScore outperforms the nine RF-Score models on all of the metrics.

**Table 4:** Performance comparison between RF-Score and DeepScore.

Model name	ROC-AUC	EF1%	EF2%	EF5%	EF10%
DeepScore	**0.98**	**52.22**	**39.72**	**18.20**	**9.47**
AV-RF-V1	0.82	29.69	21.07	11.74	7.1
AV-RF-V2	0.84	34.75	24.37	12.99	7.55
AV-RF-V3	0.84	32.72	23.04	12.6	7.47
D3.6-RF-V1	0.84	36.28	25.3	13.3	7.71
D3.6-RF-V2	0.87	43.43	29.72	14.76	8.25
D3.6-RF-V3	0.87	41.1	28.27	14.61	8.2
D6.6-RF-V1	0.77	27.42	18.65	10.37	6.42
D6.6-RF-V2	0.80	34.3	22.07	11.73	6.96
D6.6-RF-V3	0.79	32.05	21.56	11.47	6.88

Performance values of RF-Score are collected from the Supporting Information of (Wójcikowski et al., 2017). Best results are highlighted in bold.

### Sensitivity to Docking Program

Above results have shown that DeepScore works well with the docking poses generated from Glide. To examine whether DeepScore is sensitive to docking program, we regenerated all ligand poses using AutoDock Vina (Trott and Olson, 2010) and repeated the above process. ROC-EFs of test results were calculated and shown in **Tables S4** and **S5** to quantitatively assess the influence of changing docking program on the virtual screening ability of DeepScore. Obvious differences can be observed on some targets in **Table S5**. For example, DeepScore-ADV (AutoDock Vina) achieved a ROC-EF0.5% of 160.65 on HS90A which represented an improvement of 37.01% over the ROC-EF0.5% achieved by DeepScore-Glide (117.25). But on PLK1, ROC-EF0.5% dropped by 60.61 (DeepScore-ADV 84.76 *vs*. DeepScore-Glide 145.37). Generally speaking, DeepScore-ADV got a similar performance with DeepScore-Glide in terms of mean values (see **Table S4**). It can be concluded that the screening ability of DeepScore is robust and insensitive to the docking program used, on the premise that the docking program can provide reliable docking poses.

### Case Study and Visualization

An appropriate visualization method will be beneficial for lead optimization. Some deep learning–based scoring functions, like DenseFS that uses 3D CNN (Hochuli et al., 2018; Imrie et al., 2018), are rather cumbersome in explaining the results of the model. The form of DeepScore makes the interpretation and visualization of the model much more intuitive. Here, we used four targets, AA2AR, CDK2, ESR1, and DPP4, as examples to show how to visually analyze the prediction results of DeepScore. These four targets were randomly selected and belong to four different protein families: AA2AR (adenosine A2a receptor, GPCR), CDK2 (cyclin-dependent kinase 2, kinase), ESR1 (estrogen receptor alpha, nuclear receptor), and DPP4 (dipeptidyl peptidase IV, protease).

We showed the contribution of every ligand (or protein) atom to binding by coloring each atom different shades of red. Given a protein–ligand complex, the score for each atom pair could be calculated through Eq. 2-2 under a certain model. The contribution of an atom was equivalent to the sum of the scores of all atom pairs involving this atom. All of the ligand and protein atoms were initially painted dark gray. Then, atoms that contributed positively would be painted different shades of red, and the color of atoms with negative contributions would not change. The atom with the highest positive score in ligand/protein would be painted in the deepest red. The shades of the red of other atoms indicated the relative magnitude of the contribution of the atom to the contribution of the atom colored deepest red. We randomly selected a positive ligand for each target and analyzed the binding mode of the ligand to the target using above coloring strategy.

**Figure 7:**
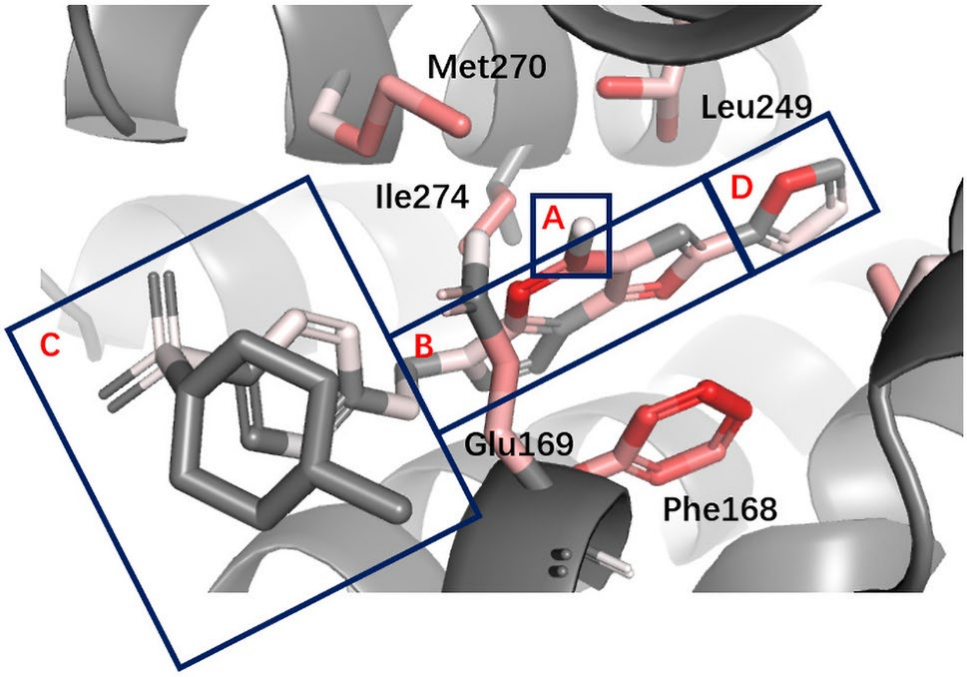
Binding mode analysis of CHEMBL418564 with AA2AR receptor (DeepScore =1.875, PDB ID 3eml). A to D refer to the four different parts in pharmacophore model of AA2AR antagonists. A, hydrogen-bond donor. B, N-containing aromatic ring. C, large lipophilic region. D, smaller lipophilic region.

**AA2AR** A2A adenosine receptors (AA2ARs) belong to G protein–coupled receptors (GPCRs). From the pharmacophore model, we have known that for AA2AR antagonists, basic structures include a hydrogen-bond donor, an N-containing aromatic ring, a large lipophilic region, and a smaller lipophilic region (Mantri et al., 2008). In **Figure 7**, the binding mode of an active obeying these pharmacophore rules is presented, and different regions are labeled. It can be seen that DeepScore highlighted the importance of the N-containing aromatic ring and the smaller lipophilic region by painting them red. The rest structures were taken as less important.

**Figure 8:**
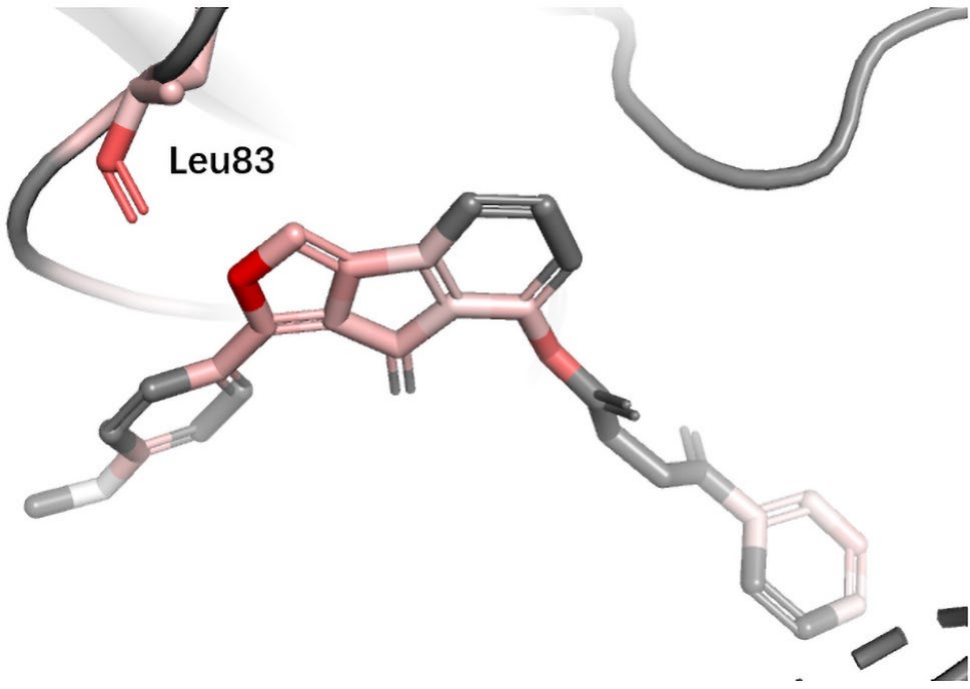
Binding mode analysis of CHEMBL363077 with CDK2 receptor (DeepScore = 1.805, PDB ID 1h00).

**CDK2** Cyclin-dependent kinases (CDKs) belong to serine/threonine family protein kinases. CDK2 is an ideal clinical target used for the treatment of breast cancer. Previous studies have shown that Leu83 residue is involved in the hydrogen bond formed with ligand (Wang et al., 2018). DeepScore also gave a high score to Leu83 and the nearest aromatic group (**Figure 8**).

**Figure 9:**
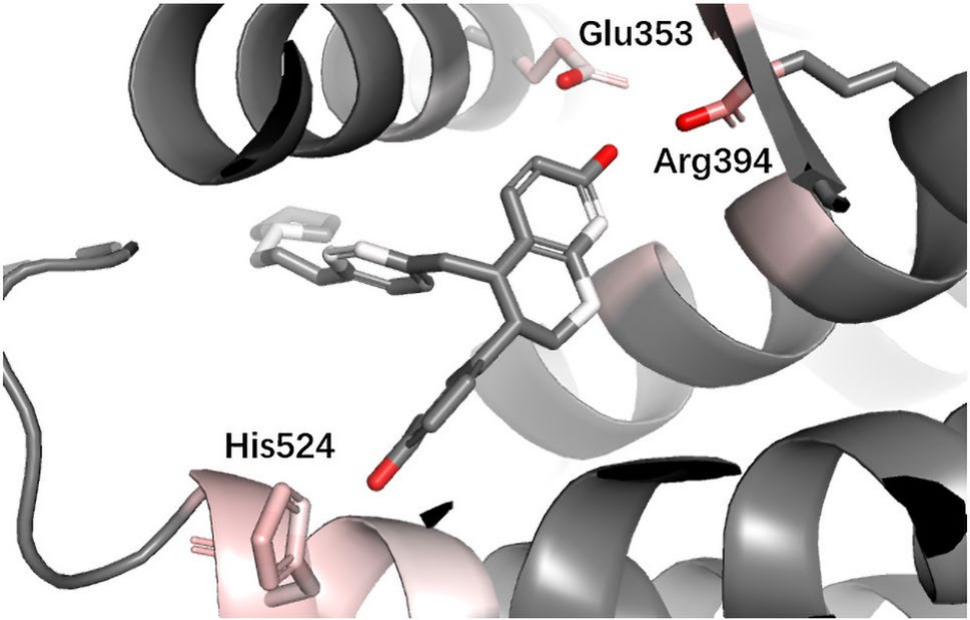
Binding mode analysis of CHEMBL56306 with ESR1 receptor (DeepScore = 7.411, PDB ID 1sj0).

**ESR1** Estrogen receptor alpha (ER alpha, ESR1) is a target for the treatment of breast cancer. Yan et al. used the information extracted by their model (PLEIC-SVM) to statistically analyze the average hydrophobic and hydrogen-bond interactions between residues of binding pocket and ligands for ESR1 (Yan et al., 2017). They found that the hydrogen bonds formed between the ligand and three residues, Glu353, Arg394, and His524, were the decisive factors in distinguishing between actives and decoy. As shown in **Figure 9**, DeepScore also ranked exact these residues as the most important three ones.

**Figure 10:**
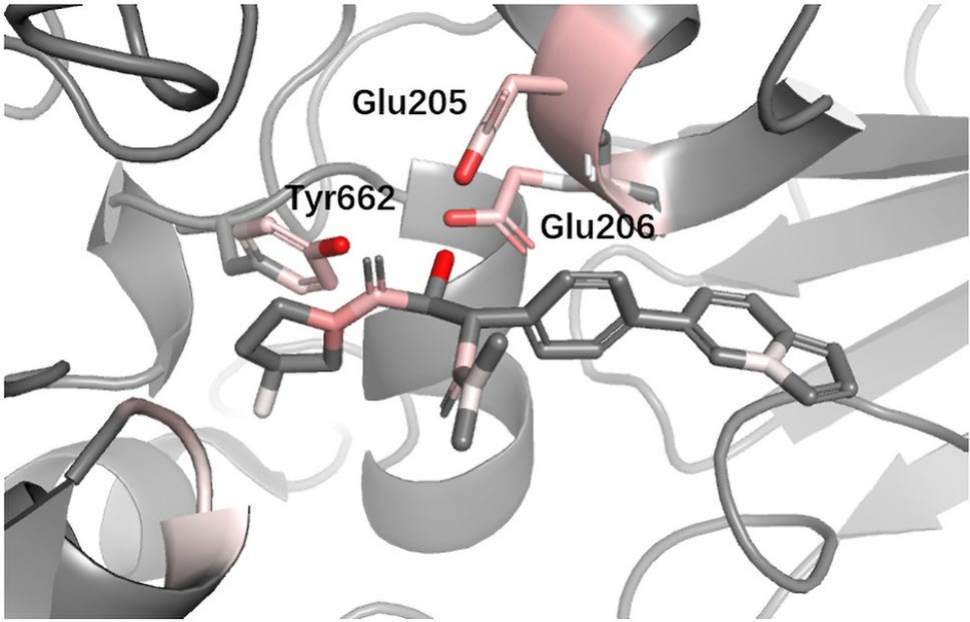
Binding mode analysis of CHEMBL378637 with DPP4 receptor (DeepScore = 3.549, PDB ID 2i78).

**DPP4** Dipeptidyl peptidase-IV (DPP4) inhibitors are used for treating diabetes mellitus. According to a recent review about DPP4 inhibitors, Glu205, Glu206, and Tyr662 in DPP4 are believed to be the most import anchor points helping inhibitors recognize DPP IV. Since we used different protein with (Ojeda-Montes et al., 2018), for the convenience of comparison, we performed sequence alignment and renumbered all residues so that the residue number we used could match (Ojeda-Montes et al., 2018). In **Figure 10**, it can be seen that DeepScore also favored these three residues and gave them fairly high scores.

## Conclusion

In this work, we introduced a novel strategy for training target-specific protein–ligand scoring functions used for structure-based virtual screening. The model outperformed Glide Gscore significantly and made progress with respect to some metrics compared with traditional machine learning–based models. These results demonstrate that our model is able to further improve the screening effect by rescoring docking poses generated from docking software. There still remains more space for improving DeepScore. Like PMF scoring function, energy terms were treated implicitly in DeepScore, which made the model more difficult to capture important protein–ligand interactions. The cutoff distance we chose may be too short, causing long-range interactions not to be captured. However, on the other side, during the experiment, we found that a larger cutoff distance would significantly increase the noise and calculation cost. The most valuable aspect of DeepScore is that it represents a novel atom-pair-based machine learning scoring strategy. With the deeper integration of deep learning and chemical informatics, we believe that deep learning–based scoring functions will further develop in the future.

## Data Availability

Publicly available datasets were analyzed in this study. This data can be found here: http://dude.docking.org/.

## Author Contributions

XL and MZ designed the study and are responsible for the integrity of the manuscript. DW, XD, and CC performed the analysis and all calculations. DW mainly wrote the manuscript. ZX contributed to data processing. HJ and KC gave conceptual advice. All authors discussed and commented on the manuscript.

## Funding

This work was supported by the National Science & Technology Major Project “Key New Drug Creation and Manufacturing Program” of China (Number:2018ZX09711002), National Natural Science Foundation of China (81573351), the Strategic Priority Research Program of the Chinese Academy of Sciences (XDA12020372), grants from Science and Technology Commission of Shanghai Municipality (18431907100), and Fudan-SIMM Joint Research Fund (FU-SIMM20174007).

## Conflict of Interest Statement

The authors declare that the research was conducted in the absence of any commercial or financial relationships that could be construed as a potential conflict of interest.
