# Supplementary material for: Improving the Virtual Screening Ability of Target-Specific Scoring Functions Using Deep Learning Methods
Source: Front Pharmacol. 2019 Aug 22;10:924. doi: 10.3389/fphar.2019.00924 (PMC6713720; doi:10.3389/fphar.2019.00924)
Supplement: Supplementary file 1 [file DataSheet_1.pdf]

Table S1. ROC-AUC and BEDROC of Gscore on DUD-E dataset

Target Name	ROC-AUC	BEDROC	Target Name	ROC-AUC	BEDROC
AA2AR	0.87	0.35	HXK4	0.62	0.36
ABL1	0.79	0.35	IGF1R	0.83	0.37
ACE	0.84	0.29	INHA	0.57	0.24
ACES	0.64	0.10	ITAL	0.67	0.16
ADA	0.76	0.45	JAK2	0.91	0.46
ADA17	0.85	0.50	KIF11	0.86	0.62
ADRB1	0.85	0.35	KIT	0.64	0.12
ADRB2	0.86	0.44	KITH	0.88	0.7
AKT1	0.69	0.28	KPCB	0.86	0.67
AKT2	0.83	0.41	LCK	0.82	0.33
ALDR	0.76	0.41	LKHA4	0.89	0.45
AMPC	0.85	0.32	MAPK2	0.95	0.64
ANDR	0.81	0.37	MCR	0.84	0.51
AOFB	0.77	0.15	MET	0.87	0.46
BACE1	0.78	0.24	MK01	0.80	0.34
BRAF	0.89	0.56	MK10	0.81	0.26
CAH2	0.63	0.06	MK14	0.70	0.31
CASP3	0.78	0.49	MMP13	0.78	0.23
CDK2	0.89	0.47	MP2k1	0.77	0.16
COMT	0.99	0.89	NOS1	0.70	0.07
CP2C9	0.61	0.00	NRAM	0.94	0.66
CP3A4	0.72	0.17	PA2GA	0.72	0.48
CSF1R	0.78	0.30	PARP1	0.92	0.61
CXCR4	0.69	0.14	PDE5A	0.86	0.41
DEF	0.91	0.24	PGH1	0.71	0.22
DHI1	0.65	0.15	PGH2	0.81	0.6
DPP4	0.88	0.21	PLK1	0.93	0.7
DRD3	0.68	0.03	PNPH	0.95	0.61
DYR	0.86	0.38	PPARA	0.82	0.24
EGFR	0.83	0.42	PPARD	0.72	0.11
ESR1	0.86	0.75	PPARG	0.85	0.4
ESR2	0.92	0.73	PRGR	0.91	0.43
FA10	0.93	0.78	PTN1	0.82	0.37
FA7	0.96	0.80	PUR2	0.99	0.99
FABP4	0.82	0.56	PYGM	0.64	0.02
FAK1	0.8	0.40	PYRD	0.86	0.61
FGFR1	0.77	0.23	RENI	0.80	0.32
FKB1A	0.88	0.68	ROCK1	0.84	0.44
FNTA	0.79	0.19	RXRA	0.97	0.81
FPPS	0.71	0.00	SAHH	0.97	0.96
GCR	0.73	0.28	SRC	0.77	0.25
GLCM	0.7	0.22	TGFR1	0.94	0.6

GRIA2	0.79	0.51	THB	0.85	0.59
GRIK1	0.87	0.33	THRB	0.91	0.59
HDAC2	0.78	0.23	TRY1	0.85	0.4
HDAC8	0.86	0.05	TRYB1	0.86	0.46
HIVINT	0.75	0.09	TYSY	0.92	0.64
HIVPR	0.77	0.27	UROK	0.85	0.63
HIVRT	0.84	0.41	VGFR2	0.72	0.34
HMDH	0.90	0.69	WEE1	1.00	0.99
HSP90	0.72	0.11	XIAP	0.92	0.81

Table S2. ROC-AUC, PRC-AUC and EF of DeepScore on DUD-E dataset

Target Name	ROC-AUC	PRC-AUC	EF0.5%	EF1%	EF2%	EF5%	EF10%
AA2AR	0.99	0.80	62.23	56.13	40.88	18.95	9.70
ABL1	0.97	0.64	45.14	40.08	34.76	17.12	8.95
ACE	0.99	0.96	60.89	60.15	47.80	19.65	9.87
ACES	0.98	0.79	62.20	59.25	38.74	18.41	9.53
ADA	1.00	0.97	59.31	58.20	48.50	19.83	9.92
ADA17	1.00	0.97	70.48	69.29	48.84	19.92	9.96
ADRB1	0.98	0.75	53.00	50.94	37.25	18.13	9.47
ADRB2	0.98	0.82	53.02	52.38	42.26	18.88	9.62
AKT1	0.99	0.89	62.37	59.03	43.96	19.48	9.86
AKT2	0.98	0.87	56.47	53.90	41.99	19.16	9.68
ALDR	0.97	0.72	51.91	47.49	35.74	16.95	9.07
AMPC	0.93	0.45	28.84	28.07	23.47	13.47	8.19
ANDR	0.97	0.67	40.00	44.76	36.64	17.80	9.46
AOFB	0.94	0.51	40.21	32.90	25.88	14.45	8.27
BACE1	0.96	0.67	54.96	50.47	34.97	17.02	9.01
BRAF	0.99	0.92	61.35	62.85	45.44	19.59	9.81
CAH2	0.99	0.90	62.35	59.52	45.65	19.21	9.76
CASP3	0.98	0.78	52.25	50.80	37.71	17.59	9.36
CDK2	0.97	0.63	46.89	43.91	34.95	17.05	9.18
COMT	1.00	0.96	93.28	87.83	49.61	20.08	10.04
CP2C9	0.87	0.28	28.21	22.04	17.36	9.77	6.41
CP3A4	0.92	0.41	41.39	37.09	27.49	14.20	7.76
CSF1R	0.98	0.72	67.59	58.96	37.86	17.52	9.46
CXCR4	0.94	0.74	73.83	56.79	38.01	18.54	9.27
DEF	1.00	0.98	58.07	58.07	49.36	19.76	9.93
DHI1	0.98	0.69	53.29	44.97	35.14	17.57	9.41
DPP4	1.00	0.96	76.80	75.47	48.53	19.76	9.95
DRD3	0.97	0.68	63.13	51.90	35.06	16.76	9.33
DYR	0.99	0.88	71.26	68.68	44.96	19.23	9.81
EGFR	0.99	0.90	63.58	61.91	45.04	19.34	9.76
ESR1	0.99	0.95	52.57	52.57	44.72	19.59	9.91
ESR2	0.99	0.95	52.73	52.73	45.37	19.32	9.74
FA10	0.99	0.92	37.13	36.75	35.63	18.92	9.70
FA7	0.99	0.95	53.24	51.48	45.25	19.69	9.94
FABP4	0.95	0.57	43.21	32.55	25.71	16.26	8.56
FAK1	0.99	0.93	54.49	53.41	44.60	19.51	9.87
FGFR1	0.97	0.68	45.94	40.91	31.10	16.64	8.78
FKB1A	1.00	0.98	52.69	52.69	46.70	20.16	10.06
FNTA	0.99	0.91	86.71	79.44	47.44	19.61	9.87
FPPS	1.00	0.97	97.52	92.83	50.66	20.03	10.01
GCR	0.97	0.47	49.84	40.91	32.07	16.23	9.35

GLCM	0.97	0.62	57.54	45.49	34.12	16.84	8.89
GRIA2	0.96	0.70	70.76	59.67	36.16	17.21	9.06
GRIK1	0.98	0.84	62.01	58.36	43.99	18.82	9.75
HDAC2	0.99	0.94	55.62	55.62	44.80	19.66	9.78
HDAC8	0.99	0.97	64.17	63.51	48.50	19.74	9.82
HIVINT	0.98	0.80	64.40	58.42	39.97	17.97	9.42
HIVPR	0.99	0.88	65.54	62.85	46.02	19.33	9.78
HIVRT	0.97	0.68	46.62	43.82	34.51	16.59	9.03
HMDH	0.99	0.86	46.53	45.01	42.84	19.73	9.87
HS90A	0.99	0.71	42.49	40.17	37.39	18.97	9.84
HXK4	0.98	0.84	49.99	47.78	36.67	18.48	9.83
IGF1R	0.97	0.71	54.12	48.02	37.46	17.79	9.19
INHA	0.96	0.79	48.48	45.93	38.00	17.07	9.60
ITAL	0.99	0.78	69.44	64.75	39.13	19.04	9.92
JAK2	0.98	0.85	60.52	56.50	41.69	18.17	9.46
KIF11	0.97	0.77	58.84	54.28	35.71	16.78	9.17
KIT	0.96	0.61	52.40	46.01	33.23	16.16	8.83
KITH	1.00	0.96	50.42	50.42	45.69	19.75	10.07
KPCB	0.97	0.61	56.12	51.17	35.87	16.66	9.21
LCK	0.98	0.79	62.18	58.14	42.09	18.54	9.52
LKHA4	0.99	0.91	40.12	39.50	36.71	19.25	9.80
MAPK2	0.98	0.82	55.15	54.13	41.36	18.69	9.44
MCR	0.98	0.60	48.70	36.38	35.28	18.52	9.84
MET	0.98	0.84	63.46	61.07	42.43	18.68	9.58
MK01	0.97	0.55	34.70	34.70	28.07	16.48	9.04
MK10	0.95	0.64	56.76	47.20	32.16	15.71	8.78
MK14	0.98	0.75	57.86	53.13	37.60	17.89	9.45
MMP13	0.99	0.95	65.46	64.93	47.06	19.44	9.81
MP2k1	0.96	0.66	53.59	54.43	37.68	16.92	9.21
NOS1	0.95	0.51	47.98	50.14	33.09	16.46	8.59
NRAM	0.98	0.93	63.71	62.62	46.69	18.90	9.66
PA2GA	0.98	0.82	49.98	47.92	37.97	18.36	9.31
PARP1	1.00	0.95	58.94	58.55	46.08	19.74	9.89
PDE5A	0.99	0.79	66.33	59.85	43.14	19.13	9.73
PGH1	0.92	0.50	43.10	36.85	22.97	13.50	7.80
PGH2	0.96	0.76	46.74	46.46	34.00	16.44	8.97
PLK1	0.97	0.76	59.32	53.69	38.54	17.22	9.27
PNPH	1.00	0.96	68.04	66.13	48.05	19.62	9.91
PPARA	0.98	0.82	42.68	41.60	33.77	18.21	9.75
PPARD	0.99	0.87	46.87	44.75	38.34	18.78	9.76
PPARG	0.97	0.78	48.69	48.05	36.46	17.43	9.28
PRGR	0.98	0.74	58.92	52.21	36.11	17.20	9.21
PTN1	0.98	0.75	49.79	45.74	38.10	18.14	9.25
PUR2	1.00	0.99	53.96	53.96	50.72	20.29	10.07

PYGM	0.95	0.49	34.72	26.77	23.54	15.17	8.90
PYRD	0.99	0.86	54.64	55.01	41.52	19.62	9.94
RENI	0.97	0.74	56.19	57.22	40.60	18.01	9.33
ROCK1	0.98	0.73	46.87	50.11	40.41	17.33	9.51
RXRA	1.00	0.95	42.44	42.44	40.21	19.40	9.94
SAHH	1.00	0.95	40.02	42.64	42.65	20.12	10.06
SRC	0.98	0.77	59.28	57.42	40.67	18.64	9.55
TGFR1	1.00	0.96	61.05	61.85	47.80	19.76	9.88
THB	0.99	0.89	41.46	42.62	41.29	19.29	9.86
THRB	0.99	0.87	57.76	56.68	43.18	18.76	9.65
TRY1	0.99	0.92	57.82	57.36	44.31	18.97	9.64
TRYB1	1.00	0.96	50.03	50.10	47.37	19.91	9.96
TYSY	0.99	0.83	59.48	54.99	40.77	18.75	9.56
UROK	0.98	0.85	58.07	56.28	42.56	18.33	9.47
VGFR2	0.99	0.83	46.98	43.98	38.26	19.08	9.86
WEE1	1.00	0.99	59.91	59.91	49.37	19.82	10.01
XIAP	1.00	0.97	51.90	50.87	47.22	19.94	9.97

Table S3. ROC-EF of DeepScore on DUD-E dataset
and Coefficient c of DeepScoreCS for different targets

Target Name	ROC-EF0.5%	ROC-EF1%	ROC-EF 2%	ROC-EF5%	ROC-EF10%	Coefficient c
AA2AR	150.10	85.03	45.33	19.25	9.71	0.84
ABL1	103.84	71.52	40.80	17.32	9.16	0.80
ACE	189.91	96.05	48.56	19.64	9.86	0.90
ACES	144.74	79.21	42.50	18.58	9.61	0.93
ADA	195.79	97.89	49.47	19.79	9.89	0.89
ADA17	193.28	97.83	49.31	19.92	9.96	0.92
ADRB1	132.40	80.28	43.44	18.36	9.51	0.83
ADRB2	166.09	86.52	45.87	19.04	9.61	0.79
AKT1	170.73	90.66	47.56	19.51	9.84	0.93
AKT2	159.13	88.08	46.99	19.14	9.66	0.85
ALDR	129.56	74.84	40.87	17.35	9.25	0.92
AMPC	58.67	45.78	28.11	13.38	8.33	0.69
ANDR	132.61	77.37	41.73	18.34	9.56	0.74
AOFB	74.78	52.17	31.74	15.30	8.52	0.80
BACE1	120.00	71.79	39.29	17.21	9.11	0.91
BRAF	181.68	94.77	48.05	19.48	9.80	0.86
CAH2	179.83	92.45	47.33	19.25	9.78	0.96
CASP3	143.51	77.82	41.43	18.08	9.49	0.97
CDK2	111.06	73.62	40.32	17.53	9.23	0.76
COMT	185.00	97.50	48.75	20.00	10.00	0.33
CP2C9	34.29	25.71	19.05	10.48	6.38	0.94
CP3A4	75.48	50.32	29.23	14.34	7.80	0.94
CSF1R	132.99	73.83	40.57	17.93	9.57	0.88
CXCR4	135.00	72.50	42.50	18.50	9.25	0.83
DEF	196.00	98.00	49.00	19.80	10.00	0.82
DHI1	118.62	74.34	41.14	18.29	9.42	0.95
DPP4	189.84	97.36	49.06	19.77	9.94	0.89
DRD3	126.32	69.89	39.26	17.43	9.39	0.87
DYR	169.75	89.65	47.19	19.49	9.79	0.85
EGFR	176.60	92.94	47.49	19.37	9.76	0.84
ESR1	183.59	95.50	48.41	19.68	9.89	0.95
ESR2	186.75	94.21	47.52	19.28	9.72	0.87
FA10	177.66	92.00	47.02	19.14	9.74	0.92
FA7	184.27	95.61	47.81	19.65	9.91	0.91
FABP4	76.44	42.44	29.56	16.93	8.69	0.58
FAK1	181.68	91.84	47.95	19.59	9.79	0.94
FGFR1	103.81	66.19	35.95	16.91	8.74	0.91
FKB1A	189.09	98.18	50.00	20.00	10.00	0.91
FNTA	183.91	94.86	48.20	19.62	9.88	0.90
FPPS	197.50	100.00	50.00	20.00	10.00	0.73
GCR	88.37	61.31	36.50	16.94	9.39	0.83

GLCM	106.18	66.91	37.27	16.84	9.00	0.73
GRIA2	130.02	71.46	39.31	17.79	9.16	0.88
GRIK1	159.68	88.95	45.97	18.79	9.70	0.75
HDAC2	177.71	93.32	48.33	19.56	9.83	0.93
HDAC8	190.15	97.56	48.78	19.63	9.88	0.91
HIVINT	148.00	80.00	42.50	18.40	9.40	0.87
HIVPR	180.24	93.80	47.38	19.34	9.77	0.90
HIVRT	126.34	73.01	39.58	16.87	9.08	0.74
HMDH	176.47	95.88	48.24	19.65	9.88	0.79
HS90A	117.25	85.03	45.36	19.29	9.76	0.74
HXK4	141.52	80.53	45.12	19.56	9.78	0.93
IGF1R	136.55	76.55	42.07	17.79	9.38	0.89
INHA	130.56	72.50	40.83	16.78	9.53	0.91
ITAL	147.27	80.00	44.55	19.45	9.91	0.92
JAK2	155.15	85.06	44.39	18.33	9.54	0.83
KIF11	134.42	73.19	38.33	17.06	9.13	0.89
KIT	112.50	65.00	36.25	17.00	8.88	0.97
KITH	182.12	94.55	47.27	19.64	10.00	0.86
KPCB	118.87	68.50	38.82	17.35	9.26	0.78
LCK	155.58	85.36	45.08	18.84	9.62	0.89
LKHA4	165.83	89.05	46.66	19.52	9.76	0.89
MAPK2	152.00	86.00	45.50	18.60	9.50	0.83
MCR	76.43	66.07	36.96	18.00	9.75	0.61
MET	161.21	85.45	45.45	18.91	9.58	0.94
MK01	89.17	52.33	31.25	16.92	9.23	0.81
MK10	104.86	62.00	36.83	16.30	8.74	0.79
MK14	136.34	78.38	42.65	18.20	9.53	0.93
MMP13	186.25	95.60	48.41	19.47	9.81	0.97
MP2k1	143.33	75.00	40.42	17.17	9.17	0.79
NOS1	99.47	64.05	35.16	16.51	8.56	0.87
NRAM	175.47	92.84	46.95	18.78	9.59	0.85
PA2GA	151.47	81.84	43.45	18.39	9.39	0.91
PARP1	185.80	96.05	48.91	19.72	9.90	0.95
PDE5A	151.67	88.73	45.84	19.19	9.73	0.85
PGH1	78.12	42.57	25.68	14.13	8.24	0.88
PGH2	133.70	72.05	39.73	16.77	9.07	0.89
PLK1	145.37	75.54	41.52	17.54	9.34	0.62
PNPH	190.19	97.05	49.02	19.80	10.00	0.82
PPARA	141.90	79.29	43.81	19.14	9.79	0.88
PPARD	159.86	85.80	44.78	19.41	9.75	0.93
PPARG	139.21	77.14	41.82	18.07	9.29	0.86
PRGR	130.32	72.70	41.06	17.93	9.29	0.78
PTN1	137.91	77.75	41.66	18.10	9.21	0.85
PUR2	192.00	100.00	50.00	20.00	10.00	0.20

PYGM	55.83	44.92	31.75	16.35	9.23	0.96
PYRD	155.44	87.19	46.78	19.58	9.89	0.90
RENI	148.00	77.86	42.76	18.45	9.42	0.88
ROCK1	149.05	81.79	42.39	17.99	9.50	0.70
RXRA	181.38	92.39	47.05	19.67	9.92	0.75
SAHH	193.33	100.00	50.00	20.00	10.00	0.35
SRC	156.44	84.00	44.90	18.73	9.58	0.83
TGFR1	189.34	97.69	48.85	19.69	9.85	0.87
THB	169.05	90.68	45.87	19.60	9.80	0.84
THRB	169.87	89.80	46.12	18.93	9.64	0.93
TRY1	177.17	91.78	46.69	19.13	9.66	0.95
TRYB1	196.00	98.67	49.67	19.87	9.93	0.98
TYSY	156.10	88.10	45.43	18.72	9.73	0.74
UROK	165.72	87.14	45.43	18.78	9.51	0.84
VGFR2	154.85	87.25	46.15	19.31	9.84	0.94
WEE1	196.00	99.00	49.50	19.80	10.00	0.32
XIAP	194.00	98.00	49.50	19.80	9.90	0.74

Table S4. Mean ROC-EF of DeepScore-Glide and DeepScore-ADV

	DeepScore-Glide	DeepScore-ADV
ROC-EF0.5%	148.95	143.32
ROC-EF1%	81.61	79.04
ROC-EF2%	43.32	42.33
ROC-EF5%	18.42	18.15
ROC-EF10%	9.50	9.41

Table S5. ROC-EFs of DeepScore-ADV on DUD-E dataset

Target	ROC-EF0.5%	ROC-EF1%	ROC-EF2%	ROC-EF5%	ROC-EF10%
AA2AR	142.24	81.94	45.01	19.04	9.65
ABL1	128.63	74.33	41.06	18.22	9.44
ACE	186.29	95.69	47.84	19.35	9.82
ACES	142.63	80.00	43.03	18.47	9.58
ADA	191.35	97.89	48.95	19.58	9.89
ADA17	192.09	96.64	49.31	19.92	9.96
ADRB1	143.76	79.33	44.22	18.93	9.75
ADRB2	157.39	87.83	46.09	19.22	9.70
AKT1	175.61	89.84	45.73	19.11	9.76
AKT2	177.54	91.38	45.69	19.14	9.74
ALDR	119.56	72.30	39.92	18.23	9.49
AMPC	52.89	43.78	25.89	13.73	7.91
ANDR	116.01	78.48	43.38	18.01	9.33
AOFB	66.09	43.48	26.09	13.91	8.52
BACE1	129.29	75.36	41.07	17.79	9.18
BRAF	173.72	90.19	46.41	18.83	9.61
CAH2	193.73	98.12	49.37	19.81	9.91
CASP3	143.59	78.85	41.69	17.88	9.19
CDK2	100.43	58.72	34.36	16.94	9.13
COMT	190.00	95.00	47.50	19.50	9.75
CP2C9	43.81	30.48	18.57	9.90	6.38
CP3A4	54.15	37.10	21.70	12.33	7.36
CSF1R	117.08	68.94	40.26	17.92	9.45
CXCR4	145.00	80.00	42.50	18.50	9.50
DEF	196.00	98.00	49.50	19.80	9.90
DHI1	116.87	69.76	39.62	17.62	9.24
DPP4	173.30	91.35	47.09	19.40	9.79
DRD3	109.05	65.26	37.79	17.18	9.09
DYR	174.91	89.63	46.55	19.40	9.79
EGFR	178.83	91.64	47.31	19.37	9.80
ESR1	181.47	94.71	48.28	19.58	9.84
ESR2	185.62	95.30	48.21	19.50	9.81
FA10	176.17	91.81	46.74	19.22	9.81
FA7	189.41	95.57	49.11	20.00	10.00
FABP4	77.33	48.67	29.56	15.69	8.67
FAK1	179.79	94.95	47.97	19.39	9.89
FGFR1	120.76	66.10	38.00	17.49	9.31
FKB1A	170.99	96.36	48.64	19.64	9.91
FNTA	177.39	92.64	47.60	19.55	9.86
FPPS	200.00	100.00	50.00	20.00	10.00
GCR	94.43	59.45	37.39	17.42	9.20
GLCM	104.73	58.18	34.09	15.64	8.80

GRIA2	122.15	67.55	37.67	17.14	8.83
GRIK1	159.68	86.95	44.97	19.19	9.59
HDAC2	172.13	90.51	46.38	18.89	9.67
HDAC8	182.84	92.63	47.24	19.14	9.69
HIVINT	134.00	79.00	42.00	18.20	9.50
HIVPR	172.49	91.47	47.19	19.34	9.71
HIVRT	107.95	66.87	37.58	16.94	8.81
HMDH	167.06	92.94	47.94	19.65	9.82
HS90A	160.65	90.72	47.09	19.29	9.76
HXK4	160.70	84.74	46.75	18.92	9.46
IGF1R	115.86	69.66	42.07	18.48	9.38
INHA	95.56	63.61	37.50	16.78	9.31
ITAL	143.64	80.00	44.09	19.09	9.82
JAK2	156.97	86.06	45.37	18.72	9.45
KIF11	120.51	65.43	35.29	15.50	8.18
KIT	130.00	73.13	40.94	18.00	9.50
KITH	178.18	94.55	49.09	19.64	10.00
KPCB	99.13	58.70	34.30	16.20	8.68
LCK	139.96	80.58	43.57	18.54	9.55
LKHA4	147.65	82.95	45.12	18.90	9.76
MAPK2	144.00	80.00	41.50	18.80	9.80
MCR	119.29	71.07	36.79	16.36	8.68
MET	144.24	80.00	42.42	18.55	9.64
MK01	71.83	50.00	33.42	16.67	8.98
MK10	73.81	42.76	27.21	13.57	7.66
MK14	123.88	73.02	41.44	18.37	9.55
MMP13	187.30	95.24	48.59	19.51	9.79
MP2k1	116.67	65.83	35.83	17.00	8.92
NOS1	91.89	61.26	35.21	16.12	8.66
NRAM	181.58	90.79	46.97	19.19	9.59
PA2GA	161.68	87.89	44.45	18.39	9.69
PARP1	181.46	94.08	48.32	19.65	9.90
PDE5A	135.06	80.68	44.50	18.98	9.81
PGH1	58.25	33.23	23.93	11.81	6.84
PGH2	131.51	71.78	38.63	16.88	8.90
PLK1	84.76	55.58	32.06	16.58	8.96
PNPH	190.48	99.05	49.52	20.00	10.00
PPARA	136.06	77.43	42.21	18.23	9.54
PPARD	154.86	87.02	46.03	19.16	9.62
PPARG	130.82	75.48	40.57	17.86	9.31
PRGR	119.84	74.54	41.27	17.55	9.25
PTN1	118.83	69.78	38.49	17.46	9.37
PUR2	168.00	86.00	46.00	19.60	9.80
PYGM	81.50	51.42	29.67	14.80	8.32

PYRD	155.20	83.04	45.23	18.95	9.89
RENI	116.67	76.71	42.24	18.06	9.42
ROCK1	118.95	72.63	42.45	18.38	9.29
RXRA	162.25	86.30	45.72	18.81	9.75
SAHH	190.00	96.67	50.00	20.00	10.00
SRC	151.05	84.01	44.99	18.69	9.58
TGFR1	187.81	96.18	48.48	19.69	9.85
THB	148.00	79.21	44.32	18.57	9.59
THRB	167.61	88.02	45.23	18.80	9.73
TRY1	173.06	89.04	46.00	19.00	9.73
TRYB1	177.20	95.33	48.33	19.60	9.93
TYSY	106.58	68.87	39.94	17.99	9.54
UROK	172.99	91.46	46.64	18.90	9.70
VGFR2	143.09	80.59	43.08	18.78	9.73
WEE1	194.00	99.00	49.50	19.80	9.90
XIAP	190.00	98.00	49.00	19.60	10.00
